# Crucial Roles of the Protein Kinases MK2 and MK3 in a Mouse Model of Glomerulonephritis

**DOI:** 10.1371/journal.pone.0054239

**Published:** 2013-01-23

**Authors:** Adam J. Guess, Rose Ayoob, Melinda Chanley, Joshua Manley, Mariana M. Cajaiba, Shipra Agrawal, Ruma Pengal, Amy L. Pyle, Brian Becknell, Jeffrey B. Kopp, Natalia Ronkina, Matthias Gaestel, Rainer Benndorf, William E. Smoyer

**Affiliations:** 1 Center for Clinical and Translational Research, The Research Institute at Nationwide Children's Hospital, Columbus, Ohio, United States of America; 2 Department of Pathology, Nationwide Children's Hospital, Columbus, Ohio, United States of America; 3 Kidney Disease Section, National Institute of Diabetes and Digestive and Kidney Diseases, National Institutes of Health, Bethesda, Maryland, United States of America; 4 Institute of Biochemistry, Hannover Medical School, Hannover, Germany; 5 Department of Pediatrics, The Ohio State University, Columbus, Ohio, United States of America; University of Florida, United States of America

## Abstract

Elevated mitogen-activated protein kinase p38 (p38 MAPK) signaling has been implicated in various experimental and human glomerulopathies, and its inhibition has proven beneficial in animal models of these diseases. p38 MAPK signaling is partially mediated through MK2 and MK3, two phylogenetically related protein kinases that are its direct substrates. The current study was designed to determine the specific roles of MK2 and MK3 in a mouse model of acute proliferative glomerulonephritis, using mice with disrupted MK2 and/or MK3 genes. We found that the absence of MK3 alone worsened the disease course and increased mortality slightly compared to wild-type mice, whereas the absence of MK2 alone exhibited no significant effect. However, in an MK3-free background, the disease course depended on the presence of MK2 in a gene dosage-dependent manner, with double knock-out mice being most susceptible to disease induction. Histological and renal functional analyses confirmed kidney damage following disease induction. Because the renal stress response plays a crucial role in kidney physiology and disease, we analyzed the stress response pattern in this disease model. We found that renal cortices of diseased mice exhibited a pronounced and specific pattern of expression and/or phosphorylation of stress proteins and other indicators of the stress response (HSPB1, HSPB6, HSPB8, CHOP, eIF2α), partially in a MK2/MK3 genotype-specific manner, and without induction of a general stress response. Similarly, the expression and activation patterns of other protein kinases downstream of p38 MAPK (MNK1, MSK1) depended partially on the MK2/MK3 genotype in this disease model. In conclusion, MK2 and MK3 together play crucial roles in the regulation of the renal stress response and in the development of glomerulonephritis, which can potentially be exploited to develop novel therapeutic approaches to treat glomerular disease.

## Introduction

Acute proliferative glomerulonephritis (APGN) typically results in reduced glomerular filtration and acute kidney injury. Several animal models have been developed to study APGN experimentally, including a mouse model in which APGN is induced by injecting an antiserum raised against mesangial cells (AMC serum) [1], [2].

The mitogen-activated protein kinase p38 (p38 MAPK) is involved in numerous signaling pathways, including cytokine signaling, which plays a role in various inflammatory and other conditions such as asthma, rheumatoid arthritis, Crohn's disease, atherosclerosis, and cancer [3]. Consequently, inhibition of p38 MAPK signaling has been developed as a new anti-inflammatory strategy [4], [5]. However, complex protein kinase interplays, feed-back effects, and side-effects of the available p38 MAPK inhibitors have all complicated this approach. Downstream targets of p38 MAPK, such as the MAPK-activated protein kinases (MK) 2 and 3 (MK2, MK3), have also attracted attention for anti-inflammatory therapeutic approaches [4], [5]. Indeed, disruption of the genes encoding MK2 and MK3 resulted in perfectly viable mice which exhibited marked resistance to endotoxic shock due to reduced proinflammatory cytokine biosynthesis [6].

Increased p38 MAPK signaling has been reported in podocytes in both human APGN, as well as in experimental models of glomerulonephritis [7]–[12]. Similarly, increased activation of p38 MAPK has been observed in various other human glomerulopathies, as well as in experimental rodent nephrosis models, and podocyte injury has been ameliorated both *in vitro* and *in vivo* using p38 MAPK inhibitors [7], [8], [13]. Given the potential benefits of inhibition of the p38 MAPK pathway, it is crucial to better understand the roles of the major downstream substrates of p38 MAPK, MK2 and MK3, in these glomerular diseases.

**Figure 1:**
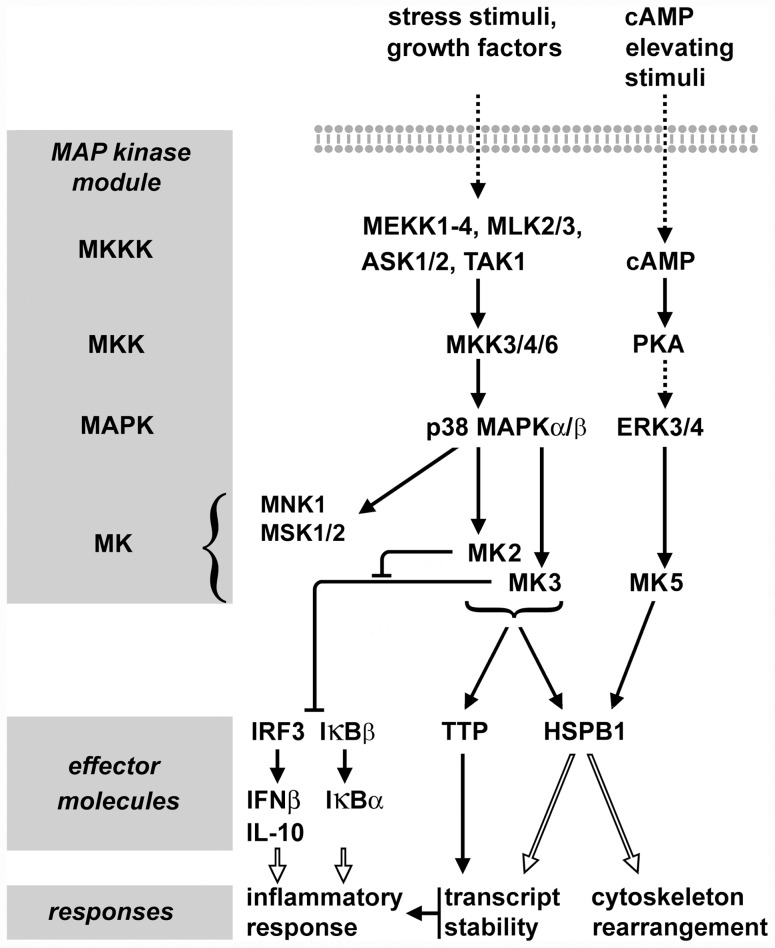
Schematic of the signal transduction involving MK2, MK3, and MK5. Activation of the p38 MAPK by various stress stimuli or growth factors results in activation of MK2 and MK3. The signal transduction by these protein kinases towards the major substrates tristetraprolin (TTP) and HSPB1 is typically additive, although in most cells MK2 is the prevailing signal transducer, with little contribution of MK3. MK5 is activated by PKA, probably independently of p38 MAPK. The role of putative MK5 becomes obvious in the absence of MK2 and MK3, as shown in the present study. MK2, MK3, and MK5 phosphorylate the same two sites in mouse HSPB1 (Ser15, Ser86). In addition to MK2 and MK3, MNK1 and MSK1/2 are further MKs that are downstream of p38 MAPK. In macrophages, MK2 and MK3 were found to control expression of the immune response mediators IFNβ, IL-10, and NFκB through regulation of the activity of IRF3 and IκBβ. In these cells, MK2 was demonstrated to prevent MK3 from exerting negative regulatory effects on IRF3- and NFκB-dependent signaling. Dashed arrows indicate indirect signal transduction, while open arrows indicate complex effects on biological responses.

MK2 and MK3 are phylogenetically closely related enzymes [14]. The presence of these two paralogous enzymes resulted from an event occurring relatively late in animal evolution, as this dualism apparently is restricted to *Amniota* (birds, mammals) with other *Bilateria* taxa (e.g. lower vertebrates) containing only one ortholog [15]. In mammals, both enzymes are ubiquitously expressed, although the expression level and activity of MK2 seems to be generally higher than that of MK3. Therefore, MK3-mediated effects can be demonstrated best in an MK2-free background [6]. Both enzymes are activated by p38 MAPK in response to identical stress factors including oxidative and osmotic stress, LPS, DNA damage, and others, and both enzymes participate in a similar, additive manner in most cellular processes studied to date, including cytokine production, gene expression, and others [6], [14]. Despite these similarities, however, recent evidence indicates that MK2 and MK3 may have different roles in LPS-treated macrophages, with MK2 regulating expression of genes like IRF3, IFNβ, IL10, IκBβ, and IκBα by preventing MK3-mediated negative effects [16]. In addition to MK2 and MK3, MAPK-interacting kinase 1 (MNK1) and mitogen- and stress-activated protein kinases 1/2 (MSK1/2) are other MKs that are downstream of p38 MAPK [17]. The relevant signal transduction events of the p38 MAPK pathway are summarized in Figure 1.

MAPK signaling networks exhibit remarkable complexity and involve multiple feedback mechanisms. For example, inactive (dephosphorylated) MK2 and MK3 form a stable complex with inactive (dephosphorylated) p38 MAPK in the nucleus [14]. Upon activation, MKK3/6 (cf. Figure 1) displaces MK2 from the complex, resulting in phosphorylation of p38 MAPK and subsequently in phosphorylation and activation of MK2. This in turn results in a conformational change of MK2 causing the export of the activated enzyme into the cytoplasm. Thus, deletion of MK2 and/or MK3 can be expected to interfere with this regulatory mechanism. In addition, deletion of MK2, or MK2 and MK3 together, resulted in a great reduction of the p38 MAPK expression level, thus affecting p38 MAPK signaling on a wider scale [14].

The small heat shock protein (sHSP) HSPB1 (HSP27, HSP25) is a major substrate of both MK2 and MK3, and its phosphorylation is frequently used to monitor the activity of these protein kinases (cf. Figure 1). Murine HSPB1 is phosphorylated by these protein kinases at Ser15 and Ser86 [18], [19]. MK5 (PRAK) is another related protein kinase that can phosphorylate HSPB1, although this enzyme seems to be regulated through the protein kinase A pathway [20], [21]. To date, no p38 MAPK stimulus is known that would activate MK5. In rodent kidneys, HSPB1 is expressed in the glomeruli, including podocytes and mesangial cells, where it plays crucial roles in cytoskeletal functions, stress response and apoptosis [22]–[25]. Induced expression of HSPB1 or other heat shock proteins, as well as components of other stress response systems, are commonly used indicators of the stress response in cells and organisms [26]. The unfolded protein response (UPR), for example, has been recognized to play a crucial role in many diseases, including nephrotic syndrome-related glomerular injury [27]–[29].

Based on the above, we hypothesized that MK2 and/or MK3 play important roles in renal injury during glomerular disease. To address this hypothesis, we analyzed the roles of these protein kinases in glomerular injury in a mouse model of APGN, using mice with disrupted MK2 and MK3 genes.

## Materials and Methods

### Animals

#### Ethics Statement

Mice were housed in animal facilities accredited by the American Association of Laboratory Animal Care, with free access to pelleted food and water. All animal experiments were conducted in accordance with the guidelines of the National Institute of Health and were approved by the Institutional Animal Care and Use Committee of the Research Institute at Nationwide Children's Hospital (AR09-00002). Mice were euthanized by inhalation of carbon dioxide in accordance with the American Veterinary Medical Association guidelines on euthanasia. Severely ill mice were sacrificed for humane reasons.

#### Mouse colony and MK2 and MK3 knock-out mice

For breeding, double heterozygote (*MK2^+/−^MK3^+/−^*) male C57BL/6 mice were crossed with wild-type C57BL/6 females (Jackson Laboratory, Bar Harbor, Maine). Offspring were genotyped and used for further breeding, eventually resulting in a sufficient number of mice with the various genotypes as included in this study: *MK2^+/+^MK3^+/+^* (wild-type), *MK2^−/−^MK3^+/+^* (MK2 knock-out), *MK2^+/+^MK3^−/−^* (MK3 knock-out), *MK2^+/−^MK3^−/−^* (MK2 heterozygote MK3 knock-out) and *MK2^−/−^MK3^−/−^* (MK2/MK3 double knock-out). More details on mouse genotyping are given in the Supporting Information (Text S1, Figure S1).

#### Model of APGN

In order to induce APGN, female mice (aged 5–7 weeks) were injected via tail veins on four consecutive days with 100 µl sheep AMC serum, as described [2]. The serum was de-complemented by heating at 56°C for 30 min before use. The numbering of experimental days began with the first dose of the AMC serum administered on day 1. Serum-treated experimental groups comprised 13 mice. At approximately day 8, a fraction of treated mice became severely ill with manifestations of hunched posture and edema that required premature sacrifice for humane reasons. These mice were included into the mortality count. Of these, kidneys from one or two mice from each group (day 8) were harvested and processed for electrophoretic and histological analysis. At day 16, one or two additional mice from the surviving fraction of each experimental group were sacrificed for tissue harvesting. Since these mice did not show any obvious manifestation of disease, they were not included into the mortality count. Controls included mice injected with de-complemented sheep pre-immune serum or were left untreated, as indicated.

### Urine and blood chemistry

Urine was collected from mice on days 0, 4, 8, 12 by applying gentle abdominal pressure. Urinary creatinine was measured with a colorimetric assay using picric acid, a modified Jaffe reaction, according to the manufacturer's recommendations (Oxford Biomedical Research, Rochester Hills, MI). Urinary protein was measured using the benzethonium chloride method as previously described [30]. Additionally, proteinuria was analyzed by sodium dodecyl sulfate polyacrylamide gel electrophoresis (SDS-PAGE) (see below).

Blood urea nitrogen (BUN) and serum creatinine were determined as described in the Supporting Information (Text S1).

### Histology and immunofluorescence microscopy

For light microscopy, kidneys were harvested on days 0, 8 and 16 after AMC serum treatment. Right kidneys were fixed in 4% paraformaldehyde and processed for paraffin embedding, while left kidneys were snap frozen in liquid nitrogen for later protein extraction (see below). Paraffin sections (2 µm) were stained with periodic acid-Schiff (PAS) stain, periodic acid-methenamine silver stain, or Masson's trichrome stain. The localization of HSPB1 in renal cortices was determined by immunofluorescence microscopy on paraffin sections [31]. In brief, sections were deparaffinized in xylene followed by rehydration in ethanol. Antigen retrieval was performed by boiling sections in solution A (10 mM sodium citrate; 0.05% Tween 20, pH 6.0). Nonspecific protein binding was blocked by incubation of the tissue sections for 1 hour in 5% control goat serum in solution B (0.3% Triton X-100 in PBS). Tissue sections were incubated overnight at 4°C with the primary rabbit anti-HSPB1 antibody (dilution: 1∶50; Enzo Lifesciences, Farmingdale, NY) in solution C (1% BSA; 0.3% Triton X-100; in PBS), followed by a 1 h incubation with the secondary FITC-coupled anti-rabbit antibody (dilution: 1∶100; Jackson Laboratory) in solution C. Immunofluorescence microscopy images were captured on a Leica DMI6000B inverted fluorescence microscope (Leica Microsystems, Bannockburn, IL) equipped with a L5 cube for visualizing FITC (excitation 480/40 nm, emission 527/30 nm). Digital micrographs were captured with a Retiga SRV 14-bit grayscale charge-coupled device camera (QImaging, Surrey, BC, Canada), and images were processed using Adobe Photoshop CS3 (Adobe Systems, Mountain View, CA).

### Protein extraction from renal cortices

Frozen kidney cortices (stored at −80°C after snap freezing) were homogenized at 4°C in tissue protein extraction reagent T-PER (Pierce, Rockford, IL) in the presence of protease and phosphatase inhibitors (Sigma-Aldrich, St. Louis, MO) at 20 µl per mg tissue, using a Dounce homogenizer. Lysates were centrifuged at 14,000× *g* for 5 min, and the supernatant was divided into aliquots and stored at −80°C. For SDS-PAGE, homogenates were mixed with 5× SDS sample buffer, yielding a final concentration of 1.0 mg/ml total protein in 62.5 mM Tris-HCl, pH 6.8; 2% SDS; 10% glycerol, 5% 2-mercaptoethanol; and 0.05% bromophenol blue. For IEF-PAGE, homogenates were adjusted to 6 M urea; 2% ampholytes 3–10; 2% Triton X-100; and 10 mM DTT (final concentrations), and containing 3.3 mg/ml total protein.

### Electrophoresis and western blotting

Electrophoretic procedures were performed according to standard protocols [32]. For SDS-PAGE analyzing tissues, 15 µl tissue extract (containing 15 µg total protein) was loaded onto 10% polyacrylamide gels. After the run, the proteins were transferred onto polyvinylidene difluoride (PVDF) membranes for immuno-labeling. For analyzing urine, samples (1 µl of urine boiled in 15 µl of SDS-sample buffer) were run on 6–20% gradient polyacrylamide gels. After the run, the gels were stained with Coomassie Brilliant Blue R250 to visualize urinary proteins.

Isoelectric focusing polyacrylamide gel electrophoresis (IEF-PAGE) was performed using a 1∶1 mixture of 3–10 and 5–7 ampholytes (BioRad, Hercules, CA). IEF-PAGE followed by western blotting was used to determine the relative distribution of the various HSPB1 isoforms (unphosphorylated, 0p; singly phosphorylated, 1p; doubly phosphorylated, 2p) within each sample, irrespective of the amounts of total HSPB1 present in the samples. Different from SDS-PAGE/western blotting, here sample loading aimed to provide similar strengths of total HSPB1 signals across all samples.

Following the electro-transfer, the PVDF membranes were incubated with primary and appropriate secondary antibodies. The following primary antibodies (Ab) were used: anti-MK2 rabbit polyclonal Ab (dilution: 1∶1000; Cell Signaling, Danvers, MA), anti-MK3 rabbit monoclonal Ab (dilution 1∶1000; Cell Signaling), anti-β-actin rabbit monoclonal Ab (dilution: 1∶5000; Cell Signaling), anti-eIF2α mouse monoclonal Ab (dilution: 1∶1000; Cell Signaling), anti-phospho-eIF2α rabbit monoclonal Ab (dilution: 1∶1000; Cell Signaling), anti-CHOP mouse monoclonal Ab (dilution: 1∶1000; Cell Signaling), anti-HSPB1 rabbit polyclonal Ab (dilution: 1∶5000; Assay Designs), anti-phospho-HSPB1-Ser86 rabbit polyclonal Ab (dilution: 1∶1000; Cell Signaling), anti-GRP78 rabbit polyclonal Ab (dilution: 1∶1000; StressMarq, Victoria, BC), anti-Hsp70 mouse monoclonal Ab (dilution: 1∶1000; StressMarq), anti-HSPB8 mouse monoclonal Ab (dilution: 1∶1000; Abcam, Cambridge, MA), anti-HSPB6 rabbit polyclonal Ab (dilution: 1∶1000; Abcam), anti-MK5 rabbit monoclonal Ab (dilution 1∶1000; Cell Signaling), anti-phospho-MK5 rabbit polyclonal Ab (dilution 1∶500; Pierce Biotechnology, Rockford, IL), anti-MNK1 rabbit monoclonal Ab (dilution 1∶1000; Cell Signaling), anti-phospho-Mnk1 polyclonal Ab (dilution 1∶1000; Cell Signaling), anti-MSK1 rabbit polyclonal Ab (dilution 1∶500; LifeSpan BioSciences, Seattle, WA), anti-phospho-MSK1 rabbit polyclonal Ab (dilution 1∶500; R&D Systems, Minneapolis, MN), and anti-GAPDH mouse monoclonal Ab (dilution 1∶5000; Millipore, Billerica, MA). Subsequently, the PVDF membranes were incubated with the appropriate horseradish peroxidase-conjugated anti-mouse or anti-rabbit IgG secondary antibodies (dilutions 1∶10,000; Jackson Laboratory, Bar Harbor, Maine, USA).

Proteins were visualized with the ECL chemiluminescence system (GE Healthcare Bio-Sciences, Piscataway, NJ) and detected by exposure to X-ray film. Representative blots from at least three independent experiments are shown.

### Statistical analysis

A 28-day survival curve was generated using the Kaplan-Meier method. The log-rank test was applied for comparison between curves. Statistical significance was defined as *P*<0.05.

The proteinuria and BUN data are presented as scatter plots that show each individual sample value, as well as the mean and standard deviation (S.D.). Statistical significance was determined by one-way ANOVA followed by Dunnett's *post hoc* testing using PRISM version 5.01 (Abacus Concepts, Berkeley, CA). Additionally, unpaired Student *t*-tests were applied to determine differences between sample groups. Probability values were considered significant at *P*<0.05.

## Results

### Deletion of MK3 or both MK2 and MK3 impairs viability of mice in a model of APGN

**Figure 2:**
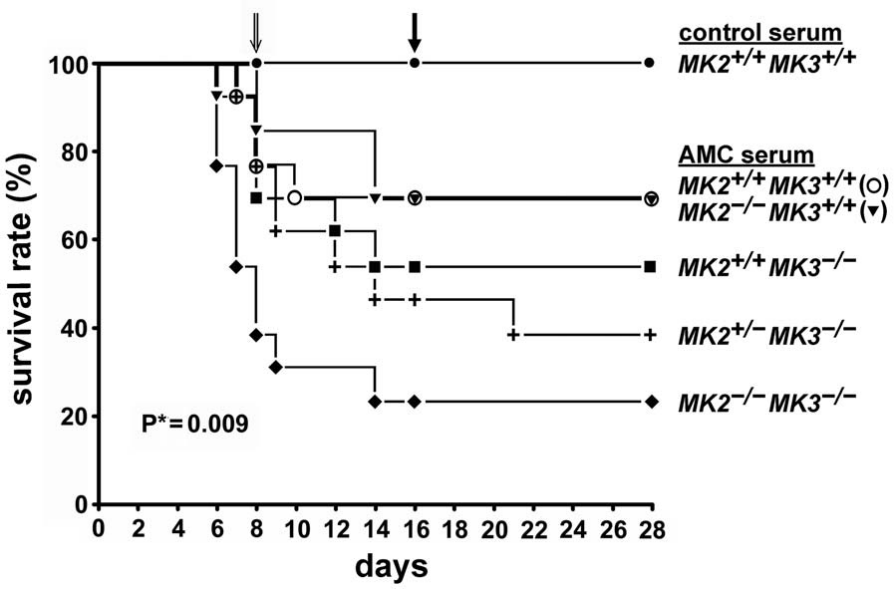
Survival rate of mice with disrupted MK2 and/or MK3 genes following injection with AMC serum. 13 mice from each group were given 100 µl of either control serum or AMC serum by i.v. injections for 4 consecutive days (days 1–4). At day 8, one or two mice from each group with pronounced disease symptoms were sacrificed for tissue harvesting (open arrow). These mice were included in the mortality count. At day 16, one or two mice from each group with no obvious disease symptoms were also sacrificed (closed arrow). These mice were excluded from the mortality count. *Log rank test.

Breeding and genotyping of mice with various MK2 and MK3 genotypes was as specified in the Materials and Methods section and in the Supporting Information (Text S1, Figure S1). Injection of AMC serum caused moderate mortality (∼31%) in wild-type mice by day 28, whereas control serum had no effect (Figure 2). As expected, the C57/BL6 mouse strain was markedly more resistant to AMC serum than the previously used FVB/N strain [2]. Deletion of MK2 (*MK2^−/−^MK3^+/+^*) resulted in similar mortality to that of wild-type mice following treatment, indicating that MK2 is dispensable for mediating resistance in the presence of MK3. Deletion of MK3 (*MK2^+/+^MK3^−/−^*) moderately increased the mortality to ∼46% at day 28, indicating a role for MK3 in the response to the AMC serum that cannot be fully compensated for by MK2. However, deletion of MK3 combined with either partial (*MK2^+/−^MK3^−/−^*) or complete (*MK2^−/−^MK3^−/−^*) absence of MK2, resulted in a further significant increase in mortality to ∼62% and ∼77%, respectively.

These data indicate that MK2 and MK3 cooperate following induction of glomerulonephritis with the AMC serum. In an MK3-free background, survival depended on MK2 in a gene dosage-dependent manner.

### Impaired renal function in response to the nephrotoxic AMC serum

**Figure 3:**
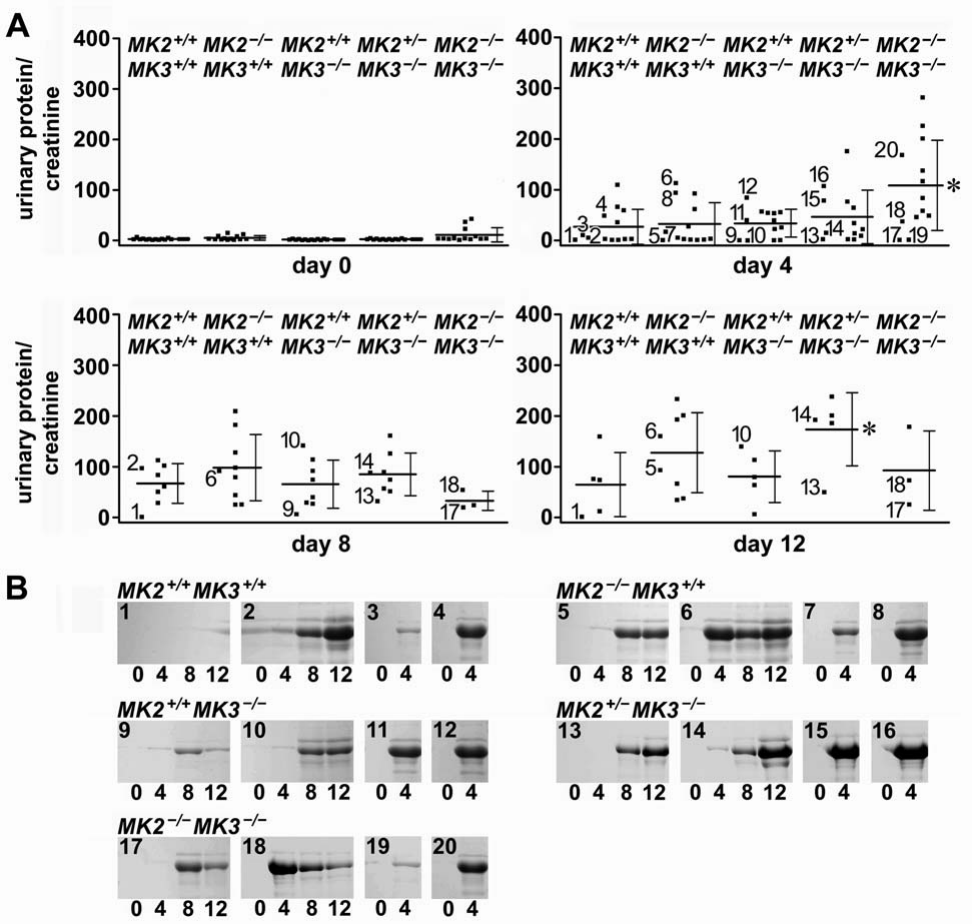
Effect of MK2 and MK3 genotypes on proteinuria in response to the AMC serum. (A) Scatter plots show the urinary protein/creatinine ratios from samples collected from all mice at day 0 prior to AMC serum injection and at days 4, 8, and 12 following AMC serum injection. Horizontal bars indicate the means and error bars represent S.D. Asterisks indicate significant (*P*<0.05) differences between means, as compared to the wild-type group at the same day. At days 8 and 12, all means were significantly greater than baseline proteinuria values at day 0 of the same genotypic group, with exception of the MK2/MK3 double knock-out (*MK2^−/−^MK3^−/−^*) mice at day 8. Given the high degree of variability within each experimental group, potential differences in mean proteinuria values among all other groups failed to reach statistical significance. Urine samples of mice selected for electrophoretic protein analysis as shown in (B) are labeled by numbers in the panels of days 4, 8, and 12. Note that in some instances the amount of collected urine was not sufficient for protein determination (i.e. the number of dots is less than the number of surviving mice as shown in Figure 2). (B) Urinary serum albumin excretion of selected mice (numbered 1 - 20) from the different *MK2* and *MK3* genotypes as visualized on Coomassie-stained SDS gels. Some of the selected mice survived throughout the entire experiment, while others died after day 4. The mouse numbers correspond to the numbered proteinuria values as indicated in (A). Consistent with the protein/creatinine ratios shown in (A), at day 0 mice of all genotypes had negligible albuminuria. Following AMC serum treatment, massive albuminuria was detected in most of the mice, with some variation in its extent and onset.

Proteinuria was analyzed as a functional indicator of glomerular injury in response to the AMC serum. At day 0, mice of all genotypes exhibited similarly low urinary protein/creatinine ratios, suggesting that deletion of MK2, MK3, or both had no major impact on baseline renal function (Figure 3A). After AMC serum treatment, proteinuria developed to a variable extent in all genotypes. Among them, MK2/MK3 double knock-out mice developed the most severe proteinuria, which peaked at day 4 and was significantly greater than in wild-type mice. This massive and early onset proteinuria correlated with the early death of most mice in this group (cf. Figure 2). Only a few surviving mice in this group exhibited mild proteinuria with delayed onset (cf. days 8 and 12). Mice of all other genotypes developed proteinuria more gradually with increases noted until day 12. With most of the double knock-out mice deceased, proteinuria at day 12 was most severe in the MK2 heterozygote/MK3 knock-out mice, and was significantly higher than in wild-type mice.

As a second indicator of renal injury, albuminuria was visualized electrophoretically for selected mice (Figure 3B). Consistent with the protein/creatinine ratios, at day 0 mice of all genotypes excreted baseline levels of serum albumin. Following AMC serum treatment, massive serum albumin excretion was detected in most mice, with some variation in its extent and onset. In general, the quantitative proteinuria data correlated well with the serum albumin detected by electrophoresis.

Renal functional injury was further evaluated by determination of BUN which is known to increase in this disease model [2]. In response to AMC serum, the average BUN values increased in mice of all genotypes with a peak at day 8 (Figure S2). This increase was statistically significant across all genotypes, further supporting the induction of functional renal injury. In addition, we determined the relative blood creatinine concentration in response to AMC serum as independent indicator of renal injury (method described in Text S1). Although we found increases in blood creatinine relative to day 0 in several individual mice, especially at day 8 following AMC serum injection, the average increases in the various experimental groups did not reach statistical significance due to the high degree of variability of the functional renal parameters observed in this disease model (results not shown).

We also analyzed changes in body weight, since renal failure may manifest as weight gain due to edema. Indeed, we observed weight gain following AMC serum injection in mice of some experimental groups (Text S1, Table S1 in Text S1). This weight gain was most pronounced (up to ∼35%) at days 8 and 12 in the MK2 heterozygote MK3 knock-out mice (*MK2^+/−^MK3^−/−^*), which correlated well with the increased proteinuria observed in this group (cf. Figure 3). The fact that the MK2/MK3 double knock-out mice (*MK2^−/−^MK3^−/−^*) which exhibited the poorest survival rate and the most severe proteinuria at day 4 did not gain weight is probably related to the early death of most of these mice before day 8. The few surviving mice in this group developed proteinuria only at a slow rate (cf. days 8 and 12 in Figure 3) which was apparently insufficient to cause weight gain.

In summary, among the different functional indicators of renal injury, proteinuria was the most responsive and informative. Collectively these functional data suggest that impaired renal function was the most likely cause of the increased mortality seen in the various MK2 and MK3 knock-out mice.

### Renal histological alterations induced by nephrotoxic AMC serum

**Figure 4:**
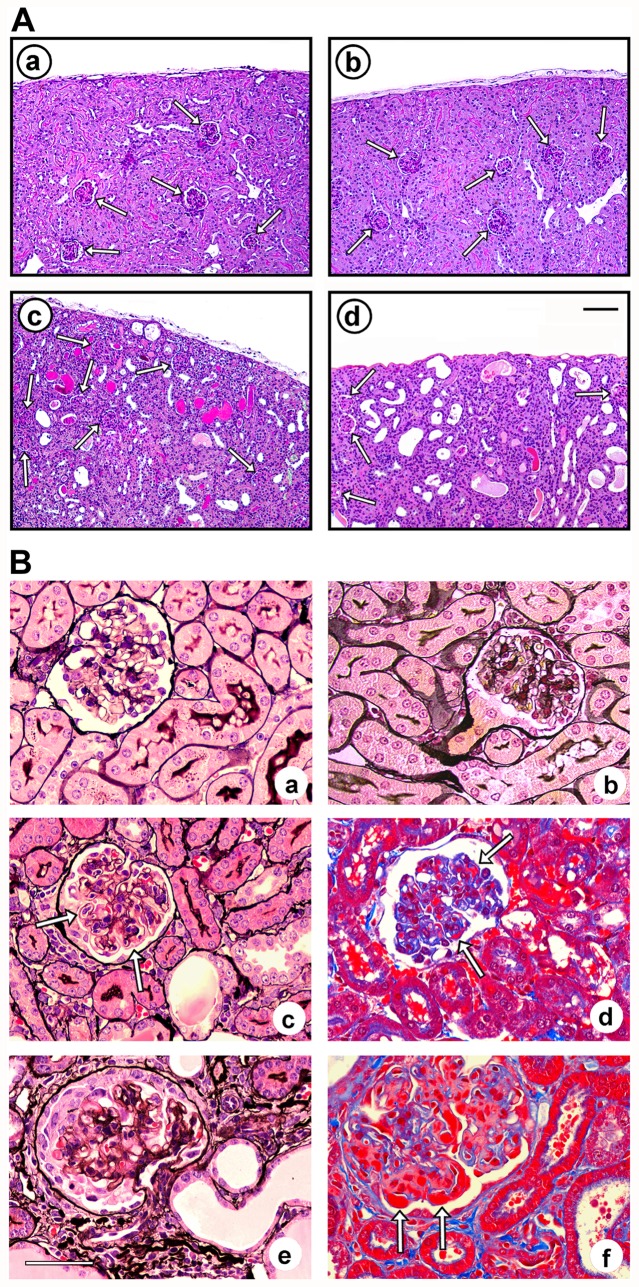
Cortical morphological lesions from wild-type and MK2/MK3 knock-out mice in response to the AMC serum. Kidney sections from untreated and AMC serum-treated wild-type mice (*MK2^+/+^MK3^+/+^*), MK2/MK3 double knock-out mice (*MK2^−/−^MK3^−/−^*), and MK3 knock-out mice (*MK2^+/+^MK3^−/−^*) on day 16 are shown. (A) Periodic acid-Schiff stain of the renal parenchyma of wild-type (panels a, c) and MK2/MK3 double knock-out mice (panels b, d), left untreated (panels a, b) or treated with the AMC serum (panels c, d). Normal morphology of the renal parenchyma was noted in untreated mice of both genotypes. Renal injury in response to the AMC serum included dilation of renal tubules and the presence of hyaline casts. Arrows designate glomeruli. Scale bar: 100 µM. (B) Silver stain (panels a–c, e) and trichrome stain (panels d, f) of glomeruli from untreated wild-type (panel a) and MK2/MK3 double knock-out mice (panel b), and from AMC serum-treated MK2/MK3 double knock-out (panels c–e) and MK3 knock-out mice (panel f). A preserved glomerular morphology was noted in untreated wild-type and MK2/MK3 double knock-out mice. Glomerular injury in response to the AMC serum included a thickening of the capillary walls due to duplication of basement membranes (tram-tracking) with associated mesangial interposition and narrowing of the capillary lumina (panel c, arrows), small fuchsinophilic subendothelial and mesangial deposits (panel d, arrows), and necrotizing lesions with associated crescent formation (panel e). These lesions were noted in mice of all genotypes. In addition, large, wire-loop type subendothelial deposits were found in the MK3 knock-out mice (panel f, arrows). Scale bar: 50 µM.

To further assess renal injury in this disease model, representative untreated control mice and AMC serum-treated mice were sacrificed at days 8 and 16, and kidneys were processed for microscopic inspection. Untreated mice of all MK2/MK3 knock-out genotypes exhibited a preserved overall renal architecture that was virtually indistinguishable from that of the control (untreated or control serum-treated) wild-type mice (Figure 4A, panel a), suggesting that neither MK2 nor MK3 is required for development of normal kidney morphology. Panel b shows the cortex from a MK2/MK3 double knock-out mouse which is representative of all other MK2/MK3 knock-out genotypes. In response to the AMC serum, renal tubules developed moderate dilation and in some cases hyaline casts, in wild-type mice (panel c), double knock-out mice (panel d), and in mice of the other genotypes (not shown), with the overall architecture of the renal parenchyma being preserved.

Prior to AMC serum treatment, the kidneys of wild-type mice (Figure 4B, panel a), MK2/MK3 double knock-out mice (panel b), and mice of the other MK2 and MK3 knock-out genotypes (not shown), all had normal glomerular morphology, suggesting that neither MK2 nor MK3 is required for glomerular morphogenesis. In contrast, on days 8 and 16 following AMC serum treatment, mice of all genotypes shared similar glomerular histopathological changes (panels c–e; shown for MK2/MK3 double knock-out mice at day 16 only). These changes included thickening of capillary walls due to reduplication of glomerular basement membranes (tram-tracking) with associated mesangial interposition resulting in narrowing of the capillary lumina (panel c). Typically, at least 50% of glomeruli were affected. Although ultrastructural studies were not performed, small fuchsinophilic deposits morphologically consistent with small fuchsinophilic subendothelial and mesangial deposits could be detected after trichrome staining in all mice (panel d). Necrotizing lesions with associated crescent formation (panel e) were observed in up to 22% of examined glomeruli at days 8 and 16 with no clear differences between the genotypes. In addition, MK3 knock-out mice showed lesions by light microscopy that were morphologically consistent with large wire-loop type subendothelial deposits (panel f).

Overall, the observed histologic lesions were similar to those previously described in the mouse strain FVB/N, although in that study no formation of crescents was reported [2]. These histological data clearly demonstrated that treated mice of all genotypes indeed developed glomerular injury in the used mouse strain.

### p38 MAPK→MK2/MK3→HSPB1 signaling and stress response in MK2 and MK3 knock-out mice following AMC serum treatment

p38 MAPK→MK2/MK3 signaling resulted in the phosphorylation of the HSPB1 in cultured podocytes [13]. In order to confirm the effects of deletion of *MK2* and/or *MK3* genes on this pathway in renal cortices, phosphorylation of HSPB1 was analyzed by two different methods: i) IEF-PAGE followed by western blotting to show the relative distribution of the various HSPB1 isoforms (0p, 1p, 2p) within each sample, and ii) SDS-PAGE followed by western blotting to show the amounts of phosphorylated HSPB1 (p-Ser86) present in each sample.

**Figure 5:**
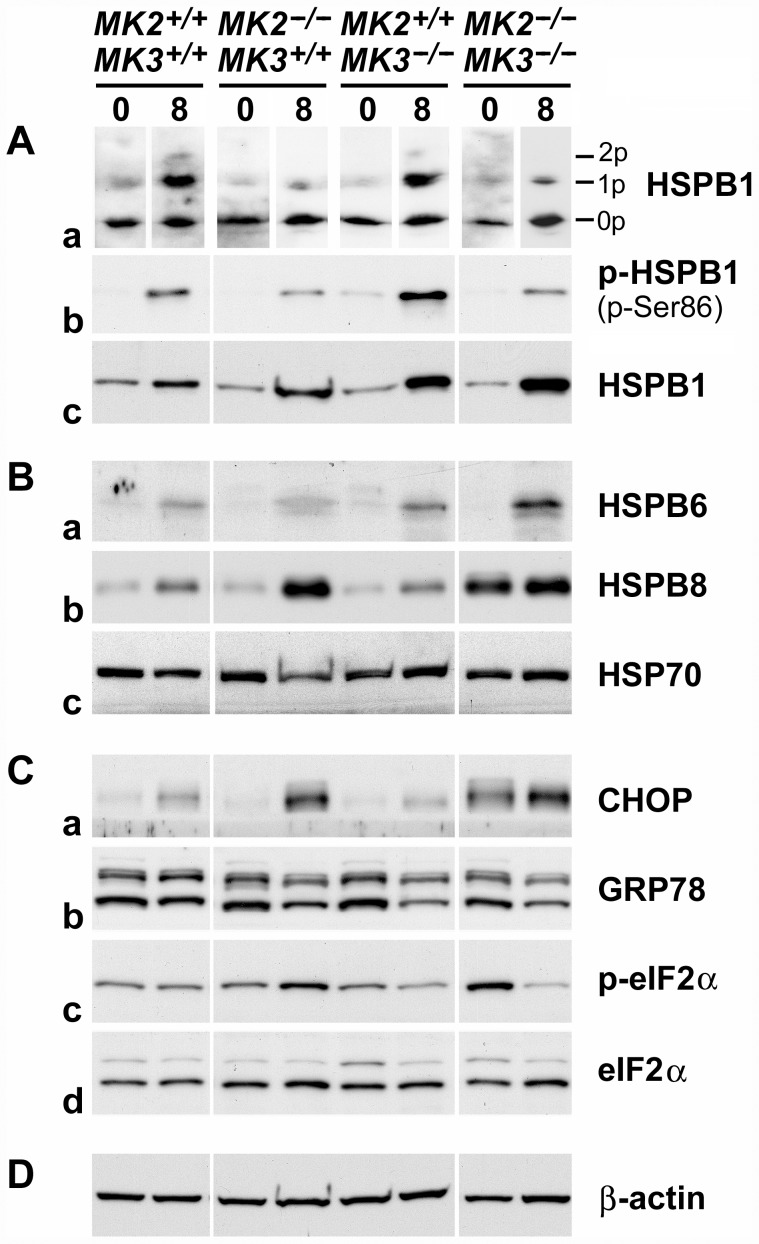
p38 MAPK→MK2/MK3→HSPB1 signaling and stress response in MK2/MK3 knock-out mice following AMC serum treatment. Extracts of renal cortices were processed for IEF-PAGE (A, panel a) or SDS-PAGE (A, panels b, c; B, C, D) from untreated mice (day 0; baseline control) and AMC serum-treated mice (day 8 of treatment). (A) Phosphorylation, baseline expression and induction of HSPB1. Panel a shows the distribution of the various HSPB1 isoforms (0p, unphosphorylated; 1p, singly phosphorylated; 2p, doubly phosphorylated) within each sample. Sample loading aimed to obtain comparable overall signals, in spite of considerable differences in the total HSPB1 content among the samples (cf. panel c). Panel b shows the amounts of Ser86-phosphorylated HSPB1 (p-Ser86). Equal amounts of total protein (15 µg) were loaded onto each lane. Panel c shows baseline expression and induction of HSPB1 in response to the AMC serum. (B) Baseline expression and response to the AMC serum of the heat shock proteins, HSPB6, HSPB8, and HSP70 (panels a–c, respectively). (C) Expression or phosphorylation of indicators of the unfolded protein response, CHOP (panel a), GRP78 (panel b), and eIF2α (panels c, d), before and after AMC serum treatment. Panels c and d show phosphorylated (p-eIF2α) and total eIF2α, respectively. (D) Expression of β-actin served as a loading control. Overall, this figure demonstrates partial involvement of MK2 and MK3 in baseline expression and/or phosphorylation of a number of sHSPs and indicators of the unfolded protein response, as well as in their pathophysiological response following AMC serum treatment.

IEF-PAGE revealed that in untreated wild-type animals, HSPB1 exists mainly in the 0p isoform, with little to no detectable 1p and 2p isoforms, respectively (Figure 5A, panel a). At day 8 following AMC serum treatment, ∼50% of HSPB1 was shifted towards the 1p isoform, and traces of the 2p isoform were detectable. As expected, a similar pattern was observed when MK3 was deleted (*MK2^+/+^MK3^−/−^*), thus confirming that MK2, rather than MK3, primarily contributed to HSPB1 phosphorylation. Consistent with this, deletion of MK2 reduced the degree of HSPB1 phosphorylation in response to the AMC serum as compared to wild-type mice, both in the presence (*MK2^−/−^MK3^+/+^*) and absence (*MK2^−/−^MK3^−/−^*) of MK3. Increased amounts of phosphorylated HSPB1 in response to the AMC serum were confirmed by SDS-PAGE using an antibody that specifically recognized phosphorylated Ser86 (panel b). The fact that residual HSPB1 phosphorylation was observed even after deletion of both MK2 and MK3 suggested the contribution of yet another protein kinase, most likely MK5 [21].

Interestingly, in addition to phosphorylation of HSPB1, we also observed a robust increase in expression of this sHSP on day 8 in response to the AMC serum (Figure 5A, panel c). Kidneys are known respond to pathophysiological stress not only with induction of ‘classic’ heat shock proteins (which include sHSPs), but also with induction or phosphorylation of indicators of the endoplasmic reticulum stress, called UPR [22], [27]–[29]. To determine the involvement of MK2 and MK3 in the stress response in this disease model, we measured the renal cortical expression and/or phosphorylation of several stress proteins and markers of the UPR. Untreated renal cortices contained similar amounts of HSPB1 regardless of the genotype (panel c). The induction of HSPB1 was strongest when MK3 was deleted (*MK2^+/+^MK3^−/−^*; *MK2^−/−^MK3^−/−^*), and seemed to correlate with the increased mortality in these genotypes (cf. Figure 2). Thus, the amount of induced renal HSPB1 seemed to indicate the extent of stress the mice experienced following AMC serum treatment. Similar expression and induction patterns were observed for two other sHSPs, HSPB6 (HSP20) and HSPB8 (HSP22) (Figure 5B, panels a and b, respectively). Renal cortices of all genotypes contained relatively low baseline levels of these sHSPs, with the exception of a high baseline level of HSPB8 in the MK2/MK3 double knock-out mice. In response to the AMC serum, induction of both HSPB6 and HSPB8 was noted in all genotypes, however, to somewhat different extents. HSPB6 was maximally induced in the absence of MK3 (*MK2^+/+^MK3^−/−^*; *MK2^−/−^MK3^−/−^*), while HSPB8 was maximally induced in the absence of MK2 (*MK2^−/−^MK3^+/+^*; *MK2^−/−^MK3^−/−^*), even though its baseline expression level was elevated in the MK2/MK3 double knock-out mice.

In order to determine if induction of these three sHSPs reflected a generalized renal cortical stress response, we also determined the expression and induction of the inducible form of HSP70 (HSP70i, HSPA1A/B). Surprisingly, expression of HSP70 remained constant across all genotypes, whether treated or untreated (Figure 5B, panel c). Thus, the induction of HSPB1, HSPB6 and HSPB8 seemed to represent a specific response to the AMC serum and did not result from a generalized stress response.

We also analyzed indicators of the UPR of the endoplasmic reticulum, including induction of the growth arrest-associated protein C/EBP homologous protein-10 (CHOP) [28] and of the endoplasmic reticulum-based chaperone glucose-regulated protein 78 (GRP78, HSPA5) [27], [29], as well as phosphorylation of the eukaryotic translation initiation factor-2α (eIF2α) [29]. Renal cortices of all genotypes exhibited low baseline expression of CHOP, except the MK2/MK3 double knock-out mice, which exhibited elevated expression (Figure 5C, panel a). CHOP was induced in all groups in response to the AMC serum. Interestingly, both the baseline expression and induction patterns of CHOP were similar to those of HSPB8, suggesting regulation by the same pathway downstream of MK2 and MK3. Baseline expression of GRP78 was similar across all genotypes (Figure 5C, panel b). However, instead of an induction, slightly decreased expression was noted in all knock-out mice in response to the AMC serum. Phosphorylation of eIF2α followed a different pattern (panel c): Baseline phosphorylation was not changed in the presence of MK2 or MK3, whereas the deletion of both MK2 and MK3 together (*MK2^−/−^MK3^−/−^*) resulted in increased phosphorylation of eIF2α. In response to the AMC serum, phosphorylation did not change in wild-type mice, as opposed to mice with deleted MK2 (*MK2^−/−^MK3^+/+^*) or MK3 (*MK2^+/+^MK3^−/−^*) which exhibited increased or decreased phosphorylation of eIF2α, respectively. Interestingly, the response to the AMC serum in the absence of both MK2 and MK3 (*MK2^−/−^MK3^−/−^*) resulted in a marked reduction in phosphorylation of eIF2α. In this situation, the absence of MK3 seemed to override the absence of MK2. For comparison, the total amount of eIF2α remained constant in these mice irrespective of the genotype or treatment (panel d).

In order to verify equal loading on the gels, β-actin was visualized on all blots throughout the experiment (Figure 5D).

**Figure 6:**
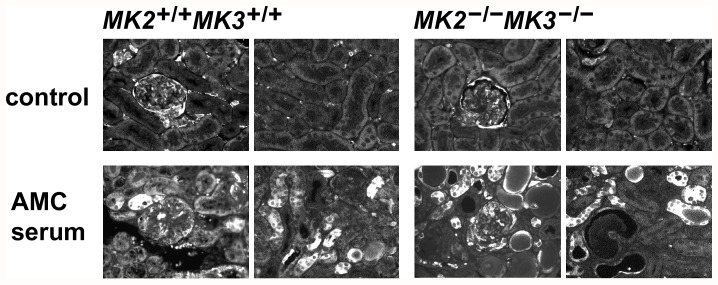
Distribution of HSPB1 in renal cortices in response to the AMC serum. Paraffin-embedded renal cortices of untreated and AMC serum-treated wild-type and MK2/MK3 double knock-out mice (day 16 following AMC serum treatment) were sectioned and processed for immunofluorescence microscopy. Total HSPB1 was visualized using an anti-HSPB1 antibody. In untreated control mice of either genotype, labeling of the glomeruli (including Bowman's space) was moderately elevated as compared to the surrounding tubules (upper row, left panels) or to the more distant tubules (upper row, right panels). AMC serum treatment caused a strong increase in HSPB1 labeling in the tubules, both adjacent to the glomeruli (lower row, left panels) and more distant from the glomeruli (lower row, right panels), thus indicating a stress response in the tubular compartment.

We also analyzed renal cortical HSPB1 induction using immunofluorescence microscopy (Figure 6). In untreated wild-type and MK2/MK3 double knock-out mice, glomeruli showed baseline HSPB1 staining (including Bowman's space), which was elevated compared to the surrounding tubules, similarly as has been described previously [22]. Following treatment with the AMC serum, a fraction of the cortical tubules exhibited strong HSPB1-positive signals in both wild-type and MK2/MK3 double knock-out mice, with only minor changes in the glomeruli, thus suggesting that the pathophysiological stress response occurred predominantly in the tubules rather than in the glomeruli.

Taken together, baseline expression and/or phosphorylation of a number of sHSPs and indicators of the UPR, as well as their pathophysiological response patterns following the AMC serum treatment, appeared to be regulated in part by MK2 and MK3. The site of the renal stress response was primarily in the tubular compartment.

### Effect of deletion of MK2 and MK3 on the expression and activation of other MKs

As mentioned above, deletion of MK2 and/or MK3 is known to affect the entire p38 MAPK signaling network through feedback mechanisms [14]. Therefore we determined the effects of MK2 and MK3 deletion in this model of APGN on the expression and activation of other p38 MAPK substrates (i.e. MNK1, MSK1) [17], in addition to MK2 and MK3 themselves, as well as the expression and activation of MK5.

**Figure 7:**
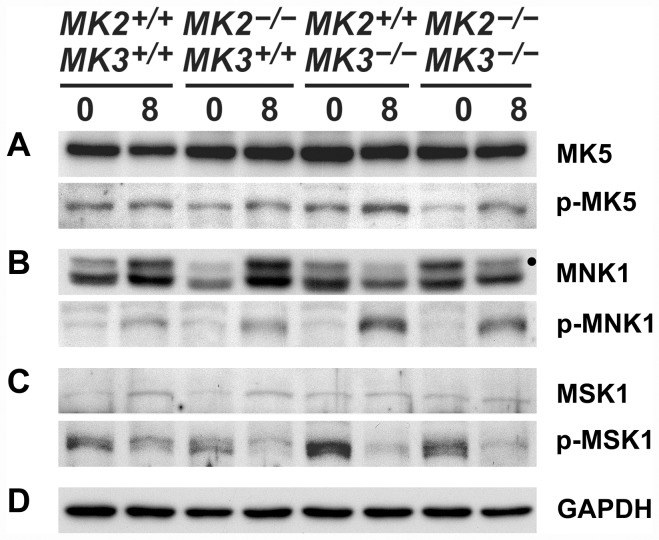
Expression and phosphorylation of various MKs in renal cortices in response to the AMC serum. Extracts of renal cortices were processed for SDS-PAGE from untreated mice (day 0; baseline control) and AMC serum-treated mice (day 8 of treatment). Expression and phosphorylation (activation) of MK5 (A), MNK1 (B), and MSK1 (C) before and after AMC serum treatment are shown. The dot in (B) marks the correct MNK1 band (upper band; ∼48 kDa), whereas the lower band (∼44 kDa) probably results from an unspecific cross-reaction of the antibody. (D) Expression of GAPDH served as a loading control.

Western blots revealed that both MK2 and MK3 were expressed in the renal cortices according to the genotype (cf. Figure S1), with no major changes following the treatment with AMC serum (not shown). MK5 was expressed at comparable levels across all genotypes, and treatment with AMC serum had no detectable effects (Figure 7A). The baseline activation (phosphorylation) of MK5 was slightly reduced in the absence of MK2 (*MK2^−/−^MK3^+/+^*, *MK2^−/−^MK3^−/−^*), while AMC serum treatment increased MK5 activation moderately in all knock-out genotypes. In the absence of both MK2 and MK3 (*MK2^−/−^MK3^−/−^*), this increased MK5 activity is reflected by the increased phosphorylation of HSPB1 seen in response to the AMC serum (cf. Figure 5A, panels a, b).

Baseline expression of MNK1 depended somewhat on both MK2 and MK3 expression, although with disparate consequences (Figure 7B): The absence of MK2 alone (*MK2^−/−^MK3^+/+^*) reduced MNK1 expression, whereas the absence of MK3 alone (*MK2^+/+^MK3^−/−^*) slightly increased its expression. The absence of both MK2 and MK3 (*MK2^−/−^MK3^−/−^*), however, resulted in a greatly increased baseline MNK1 expression. Following treatment with AMC serum, a strong induction of MNK1 was observed in the presence of MK3 only (*MK2^+/+^MK3^+/+^*, *MK2^−/−^MK3^+/+^*), in contrast to the decreased MNK1 expression observed in the absence of MK3 (*MK2^+/+^MK3^−/−^*, *MK2^−/−^MK3^−/−^*). This pattern of MNK1 expression was in contrast to its pattern of activation (phosphorylation) in response to AMC serum. The activation of MNK1 in all genotypes implied that this was largely independent of the MK2/MK3 genotypes, although it was more pronounced in the absence of MK3 (*MK2^+/+^MK3^−/−^*, *MK2^−/−^MK3^−/−^*). For comparison, the extent of baseline MNK1 activation was independent of the MK2/MK3 genotype. This behavior of MNK1 is noteworthy, since its AMC serum-induced activation was in direct contrast to its simultaneously reduced expression in the absence of MK3 (*MK2^+/+^MK3^−/−^*, *MK2^−/−^MK3^−/−^*). In summary, both MK2 and MK3 have pronounced effects on the expression and degree of activation of MNK1, both before and after induction of APGN.

In contrast to MNK1, baseline MSK1 expression was essentially constant in all genotypes, with only a slight induction noted in response to AMC serum in the presence of MK3 (*MK2^+/+^MK3^+/+^*, *MK2^−/−^MK3^+/+^*) (Figure 7C). Conversely, baseline activation (phosphorylation) of MSK1 was greatly increased in the absence of MK3 (*MK2^+/+^MK3^−/−^*, *MK2^−/−^MK3^−/−^*), whereas MSK1 activation in response to AMC serum decreased in all genotypes, suggesting that this effect is not regulated by MK2 or MK3. Similar to MNK1, MSK1 also showed a disparate pattern of expression and activation, although the specific regulation of these two protein kinases was different. In summary, MK3 has an apparent inhibitory effect on baseline MSK1 activation.

In order to verify equal loading on the gels, GAPDH was visualized for all samples (Figure 7D).

## Discussion

Manipulation of p38 MAPK signaling is emerging as an auspicious novel therapeutic strategy in inflammatory and other disorders, including NS. Our group recently demonstrated that a MK2 inhibitor protected cultured podocytes from an injury that mimics NS, similar to that reported for a p38 MAPK inhibitor [13]. However, the relationship between p38 MAPK and its downstream signaling targets is not straightforward and as a result is not well understood. Complicating factors include feedback mechanisms and redundant functions of MK2, MK3, and potentially of other protein kinases. In order to define the role of MK2 and MK3, the use of single and double gene knock-out mice are highly advantageous. Given the established role of p38 MAPK signaling in glomerular function and injury [7]–[12], [33], this study was thus designed to determine the roles of p38 MAPK's immediate downstream protein kinases MK2 and MK3 in a mouse model of APGN.

Our mortality data suggest that MK2 and MK3 together play crucial roles in the development of glomerulonephritis, with both MKs exerting partially redundant but also specific functions. Since the MK2 knock-out mice (*MK2^−/−^MK3^+/+^*) exhibited no apparent phenotype, it can be assumed that MK3 fully replaces the lost functions of MK2. The fact that the MK3 knock-out mice (*MK2^+/+^MK3^−/−^*) performed poorer than the wild type mice suggests that MK2 apparently cannot completely compensate for the loss of MK3, which may indicate that MK3 has a specific function. However, to a certain degree MK2 can compensate for the loss of MK3, which was obvious when the different MK2 genotypes (*MK2^+/+^*, *MK2^+/−^*, *MK2^−/−^*) were compared in an MK3-free background. Consistent with this, MK2/MK3 double knock-out mice (*MK2^−/−^MK3^−/−^*) exhibited the poorest performance.

In our study we observed a relatively high degree of variability with respect to the observed proteinuria (cf. Figure 3) and BUN values (cf. Figure S2), or of other parameters (histological findings, blood creatinine, body weight; cf. Figure 4, Text S1) which obscured, in part, differences between the various genotypes. Such variability, however, seems to be inherent not only to this model of renal disease [2], but also to other models including diabetic nephropathy [34], puromycin aminonucleoside rats [35], or Heymann nephritis [36]. Studies with significantly larger animal numbers would be required to overcome such variability. In spite of the limitations of our study, we determined clear effects of MK2 and MK3 on both the overall survival and molecular responses (stress response, MK expression and activation) in this model of glomerulonephritis.

The renal stress response systems, including the UPR, play crucial roles in kidney physiology and disease [22], [27]–[29], [37]. In this context, we analyzed the expression and/or phosphorylation of several stress proteins and other indicators of renal stress response, using the degree of phosphorylation of HSPB1 as an activity indicator of the upstream signaling via p38 MAPK, MK2 and MK3, and also of the alternative signaling by the putative MK5 [13], [14], [21]. Interestingly, no major change in baseline HSPB1 phosphorylation was noted in the various MK2 and MK3 genotypes, suggesting that neither MK2 nor MK3 activity was crucial for the homeostasis observed in control conditions (Figure 5A, panel a). Similarly, baseline HSPB1 and HSPB6 expression were not regulated to a detectable level by MK2 or MK3 (Figure 5A, panel c; B, panel a). In contrast, baseline expression of HSPB8 and CHOP, which also did not differ in the various MK2 and MK3 genotypes in the presence of at least MK2 or MK3, exhibited a strong increase in the MK2/MK3 double knock-out mice (Figure 5B, panel b; C, panel a). Thus, absence of both MK2 and MK3 apparently released a suppression that resulted in increased HSPB8 and CHOP expression. Similarly, suppressed baseline eIF2α phosphorylation was released in the absence of both MK2 and MK3 (Figure 5C, panel c; compared with the total amount of expressed eIF2α shown in panel d). These effects of MK2 and MK3 on baseline expression or phosphorylation of renal stress indicators are summarized in Table 1. In contrast to these striking changes, other indicators of a generalized stress response, including baseline expression of HSP70 and GRP78, were not affected by the absence of MK2 or MK3, suggesting separate regulatory pathways (Figure 5B, panel c; C, panel b).

**Table 1:** Summary of the observed baseline regulation in renal cortices with various MK2 and MK3 genotypes.

Response	Protein	I. MK2	II. MK3	III. MK2/3
Expression	HSPB8	-	-	↓
	CHOP	-	-	↓
	MNK1	↑	(↓)^1^	-
Phosphorylation	eIF2α	-	-	↓
	MK5	-	-	↑
	MSK1	-	↓	-

1 The negative effect of MK3 on MNK1 expression was inhibited by MK2.

MK2 and/or MK3 altered expression of HSPB8, CHOP, and MNK1, and phosphorylation of eIF2α, MK5, and MSK1, in the indicated manner. Positive (↑) and negative (↓) effects of MK2 and/or MK3 on expression or phosphorylation of various stress indicator proteins and MKs are indicated by the corresponding arrows, and weak responses are indicated by parentheses. Responses that apparently depended on the action of MK2 or MK3 alone are indicated in columns I and II, respectively. Responses that seemed to involve both MK2 and MK3, are indicated in column III.

Following induction of glomerulonephritis, distinct regulatory mechanisms became evident at day 8 that involved both MK2 and MK3, and probably MK5 to a minor degree. As expected, MK2 was the major mediator of HSPB1 phosphorylation, with minor participation by MK3 and putative MK5 (Figure 5A, panels a, b). The finding that all three analyzed sHSPs were induced in all genotypes suggested a common regulatory system, which was specific for these sHSPs, but not for HSP70 expression, which remained constant. However, notable differences in the induction patterns were also observed, e.g. between HSPB1 compared to HSPB8 and CHOP. Compared to wild-type mice, HSPB1 induction was greater in the absence of either MK2 or MK3, and was strongest in the absence of both kinases (panel c). This tentatively suggests additive effects of deficient MK2 and MK3 in releasing an apparent suppression of HSPB1 expression. Induction of HSPB6 may follow a similar pattern, although this was less clear. In contrast, induction of HSPB8 and CHOP was controlled differently, with MK2 being the prevailing regulator, and without detectable effects of MK3 (Figure 5B, panel b; C, panel a). Thus, the absence of MK2 seems to be sufficient for the release of suppression of HSPB8 and CHOP expression. Finally, eIF2α phosphorylation in response to disease induction followed yet a different pattern, with increased or decreased phosphorylation depending on the specific MK2/MK3 genotype (Figure 5C, panel c in comparison to the total amount of expressed eIF2α shown in panel d). A plausible explanation for this is that MK3 promotes phosphorylation of eIF2α (without eIF2α being a direct substrate of MK3), whereas MK2 inhibits this function of MK3, similar to that reported for the regulation of IRF3, IFNβ, IL10, IκBβ, and IκBα in macrophages where MK2 also prevented MK3 from exerting negative regulatory effects [16] (cf. Figure 1). The observed effects of MK2 and MK3 on the expression or phosphorylation of indicators of renal stress in response to AMC serum are summarized in Table 2. Taken together, the induction of APGN in this disease model involves the activation of specific components of the renal stress response systems that depend, in part, on MK2 and MK3.

**Table 2:** Summary of the observed regulation in response to the AMC serum in renal cortices with various MK2 and MK3 genotypes.

Response	Protein	I. MK2	II. MK3	III. MK2/3
Expression	HSPB1	-	-	↓
	HSPB6	-	-	↓
	HSPB8	↓	-	-
	CHOP	↓	-	-
	MNK1	-	↑	-
Phosphorylation	HSPB1	-	-	↑
	eIF2α	↓	↑^1^	-
	MNK1	-	↓	-

1 The positive effect of MK3 on eIF2α phosphorylation was inhibited by MK2.

MK2 and/or MK3 altered expression of HSPB1, HSPB6, HSPB8, CHOP, and MNK1, and phosphorylation of HSPB1, eIF2α, and MNK1, in the indicated manner. See Table 1 for further explanation.

Although p38 MAPK signaling includes housekeeping functions, activation of this signaling pathway is also part of the stress response system [3]. Given the known complex regulatory patterns of this pathway, we analyzed possible consequences of MK2 and MK3 deletion on expression and activation (phosphorylation) of other MKs, including MNK1 and MSK1, which are also direct substrates of p38 MAPK [17], and MK5. Similar to the regulation of the various stress response components described above, we found disparate effects of MK2 and MK3 on both the expression and activation of MNK1, MSK1, and MK5. For example, baseline MNK1 expression was subject to clear cooperation of both MK2 and MK3, with MK2 promoting MNK1 expression and, at the same time, inhibiting the negative effect of MK3 (cf. Figure 7B). In contrast, expression of MSK1 (cf. Figure 7C) and MK5 (cf. Figure 7A) were less, or not at all, affected by the presence of MK2 or MK3. More surprising, however, was the disparate regulation of the activation (phosphorylation) of MNK1 and MSK1 by MK2 and/or MK3 (cf. Figure 7 B, C), since both MNK1 and MSK1 are direct substrates of p38 MAPK. This was especially impressive with regard to the opposing responses of MNK1 and MSK1 following AMC serum treatment in the absence of MK3 (*MK2^+/+^MK3^−/−^*, *MK2^−/−^MK3^−/−^*) where activation of MNK1 was accompanied by deactivation of MSK1.

A surprising finding was the involvement of another protein kinase, in addition to MK2 and MK3, in HSPB1 phosphorylation. The best known candidate protein kinase is MK5, which phosphorylates HSPB1 at identical sites to that of MK2 and MK3 [17], [20]. In the absence of both MK2 and MK3, MK5 was activated in the renal cortices in response to AMC serum (c.f. Figure 7A), and this activation was consistent with the observed increase in HSPB1 phosphorylation (cf. Figure 5A, panels a, b). MK5 is subject to regulation by the cAMP pathway independent of the p38 MAPK pathway (Figure 1) [21]. Interestingly, activation of the cAMP-pathway suppresses experimental mesangial proliferative glomerulonephritis [38], and, hence, MK5-dependent phosphorylation may contribute to that. In this context it is of interest that the MK2 inhibitor C23, which has been shown to protect podocytes from PAN-induced injury, inhibits MK5 with a similar IC_50_ as that for MK2, but is otherwise highly specific since it did not affect ∼200 other tested protein kinases [13], [39]. This situation with partially redundant protein kinases may have important consequences for the future development of clinical therapies based on inhibition of the p38 MAPK pathway.

The fact that we observed increased HSPB1 phosphorylation in some settings (cf. Figure 5) but no corresponding activation of MK2 or MK3 (not shown) is not a contradiction. In addition to the role of MK5, the assays used may also contribute to this phenomenon. HSPB1 phosphorylation is a far more sensitive indicator of the activities of upstream protein kinases compared to the phospho-isoforms of the protein kinases themselves, since the signal gets integrated over time at the level of HSPB1. Accordingly, a slightly higher degree of MK2 activation, which may be undetectable, can result in the noticeable accumulation of phosphorylated HSPB1 over the course of 8 days. In addition, the overall degree of MK2/MK3 activation was low in the renal cortices in our study, compared to other experimental systems [13].

The observed effects of MK2 and MK3 on the expression or activation of the various MKs, both at baseline and in response to AMC serum, are also summarized in Tables 1 and 2, respectively. These regulatory patterns are consistent with the known complex nature of p38 MAPK signaling. Part of the observed alterations in expression of indicators of the stress response or MKs likely resulted from modulated activities of transcription factors, since MK2 and MK3 phosphorylate a number of them [15]. Similarly, p38 MAPK phosphorylates transcription factors [40], and the secondary down-regulation of p38 MAPK due to deletion of MK2/MK3 can also be expected to affect their activities. Such mechanisms may also underlie the regulated expression of HSPB1, HSPB8, CHOP, or MNK1 in the renal cortices. Increased phosphorylation in the absence of MK2 and/or MK3, whether at baseline or following the induction of APGN, may result from a re-direction of the signal towards other p38 MAPK substrates such as MNK1 and MSK1. Alternatively, in the absence of MK2 and MK3 the signal may circumvent the p38 MAPK pathway altogether and instead use the ERK1/2 pathway, which also activates MNK1 and MSK1 [17]. Unraveling this complexity of MAPK signaling in renal disease will clearly require further studies.

The group with the highest mortality (MK2/MK3 double knock-out mice) exhibited several characteristic abnormal patterns of expression and/or phosphorylation of the stress response indicators and of the MKs studied, in baseline conditions and/or following disease induction. These aberrant responses included expression of HSPB8, CHOP, and MNK1, and phosphorylation of eIF2α and MK5 (cf. Figures 5, 7). We expect that some of these abnormal responses may have contributed to the severity of APGN in these double knock-out mice.

In recent years inappropriately activated signaling pathways in podocytes or other renal cells have been recognized as causes for renal injury. Examples include increased Notch signaling in the podocytes of patients with glomerular proteinuria [41], the protective effects of down-regulated PKCα in a mouse model of diabetic nephropathy [42], the protective effects of inhibition of p38 MAPK in animal models of renal disease [7], [8], and inappropriate mTOR signaling in podocytes [43]. However, the study of mTOR also showed that both signaling and its inhibition are ambivalent and context-dependent, and a better understanding of the signaling network will be needed to enable the design of a mTOR-targeted therapy for glomerular disease [43]. A similar situation may be the case for targeting the p38 MAPK→MK2/MK3 signaling network. Notably, in our study deletion of MK2 alone did not exacerbate the disease as long as MK3 was present, compared to wild-type mice. In fact, deletion of MK2 may even have resulted in a slight improvement in the survival at day 10 (cf. Figure 2). This suggests the possibility that controlled and partial inhibition of MK2 activity (i.e. pharmacologic inhibition vs. complete genetic deletion) may be required to optimize the potential clinical benefits of reduced MK2 activity. Perhaps the most surprising and important insight from this study was that for future therapeutic approaches, preservation of MK3 activity seems to be critical.

In summary, our study found that MK2 and MK3 play critical, interconnected roles in the regulation of the development of glomerulonephritis and the renal stress response. These data also support the concept that partial and selective inhibition of MK2 represents an attractive potential therapeutic approach for the treatment of glomerular disease. However, further understanding of this pathway and the interactions among its members is needed to optimize the benefits of such an approach.

## Supporting Information

Figure S1 **MK2 and MK3 knock-out genotypes of C57/BL6 mice as used in this study.** (A) PCR genotyping using allele-specific primers. The positions of PCR products specific for wild-type (wt) and knock-out (ko) alleles of MK2 and MK3 are indicated on the right. The two leftmost lanes show molecular mass markers with the positions of 1000 and 300 bp indicated (bars). (B) Expression of MK2 and MK3 in mice with different MK2 and MK3 genotypes, as shown by western blotting and using an MK2- and MK3-specific antibodies.
(TIF)

Figure S2 **Effect of MK2 and MK3 genotypes on BUN in response to the AMC serum.** The BUN values collected at day 0 prior to AMC serum injection and at days 4, 8, and 12 following AMC serum injection were plotted for each surviving mouse. Horizontal bars indicate the means and error bars represent S.D. At days 8 and 12, all means were significantly different from the baseline values at day 0 of the same genotype group. The trend of BUN values was consistent with the proteinuria data, with the MK2/MK3 double knock-out mice being most susceptible to injury.
(TIF)

Text S1 **Additional information on mouse genotyping and determinations of blood urea nitrogen, blood creatinine and body weight.**
(DOC)
