# Supplementary figures and images for: Crucial Roles of the Protein Kinases MK2 and MK3 in a Mouse Model of Glomerulonephritis
Source: PLoS One. 2013 Jan 23;8(1):e54239. doi: 10.1371/journal.pone.0054239 (PMC3553169; doi:10.1371/journal.pone.0054239)

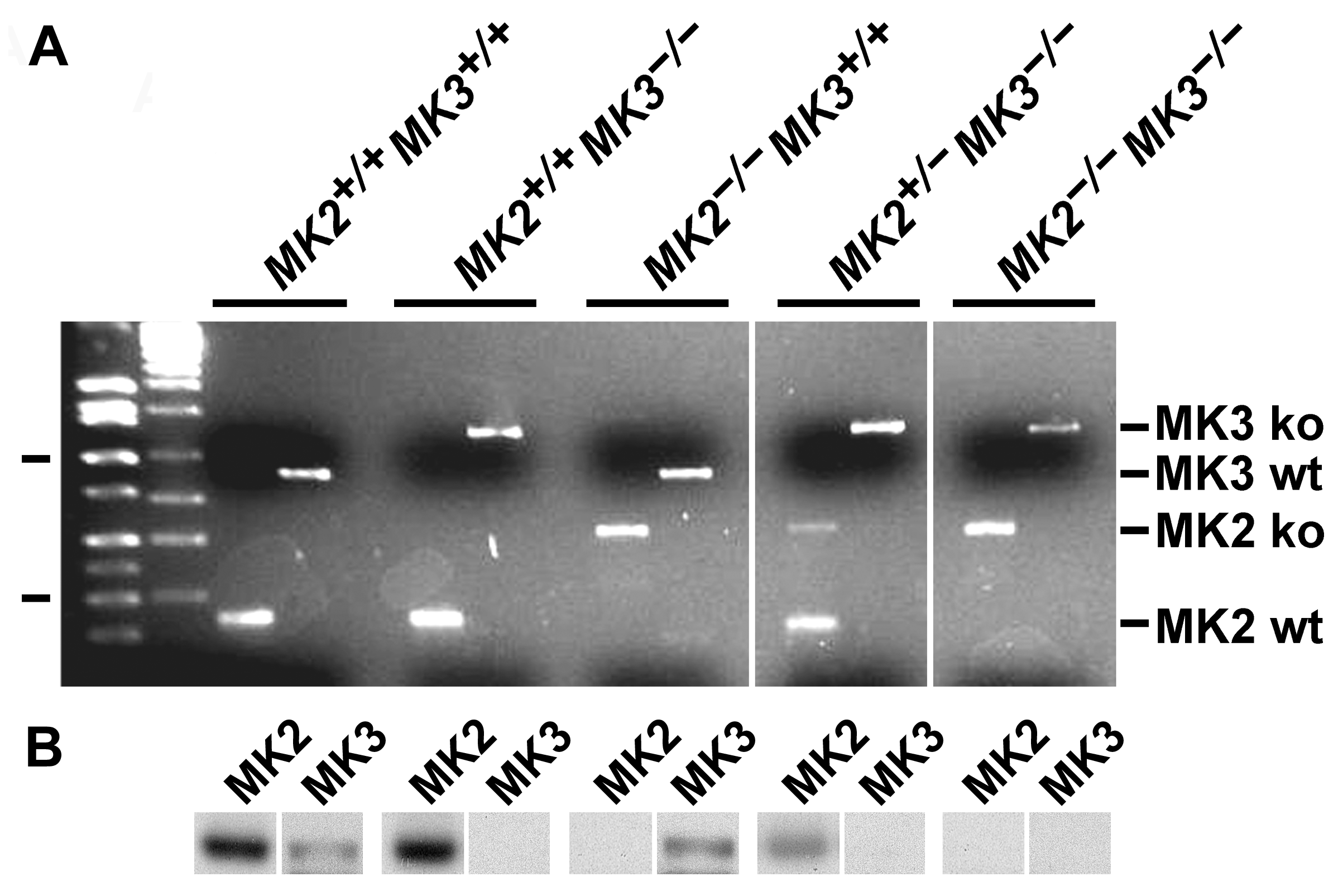

Supplement: Figure S1 — MK2 and MK3 knock-out genotypes of C57/BL6 mice as used in this study. (A) PCR genotyping using allele-specific primers. The positions of PCR products specific for wild-type (wt) and knock-out (ko) alleles of MK2 and MK3 are indicated on the right. The two leftmost lanes show molecular mass markers with the positions of 1000 and 300 bp indicated (bars). (B) Expression of MK2 and MK3 in mice with different MK2 and MK3 genotypes, as shown by western blotting and using an MK2- and MK3-specific antibodies. (TIF) [file pone.0054239.s001.tif]

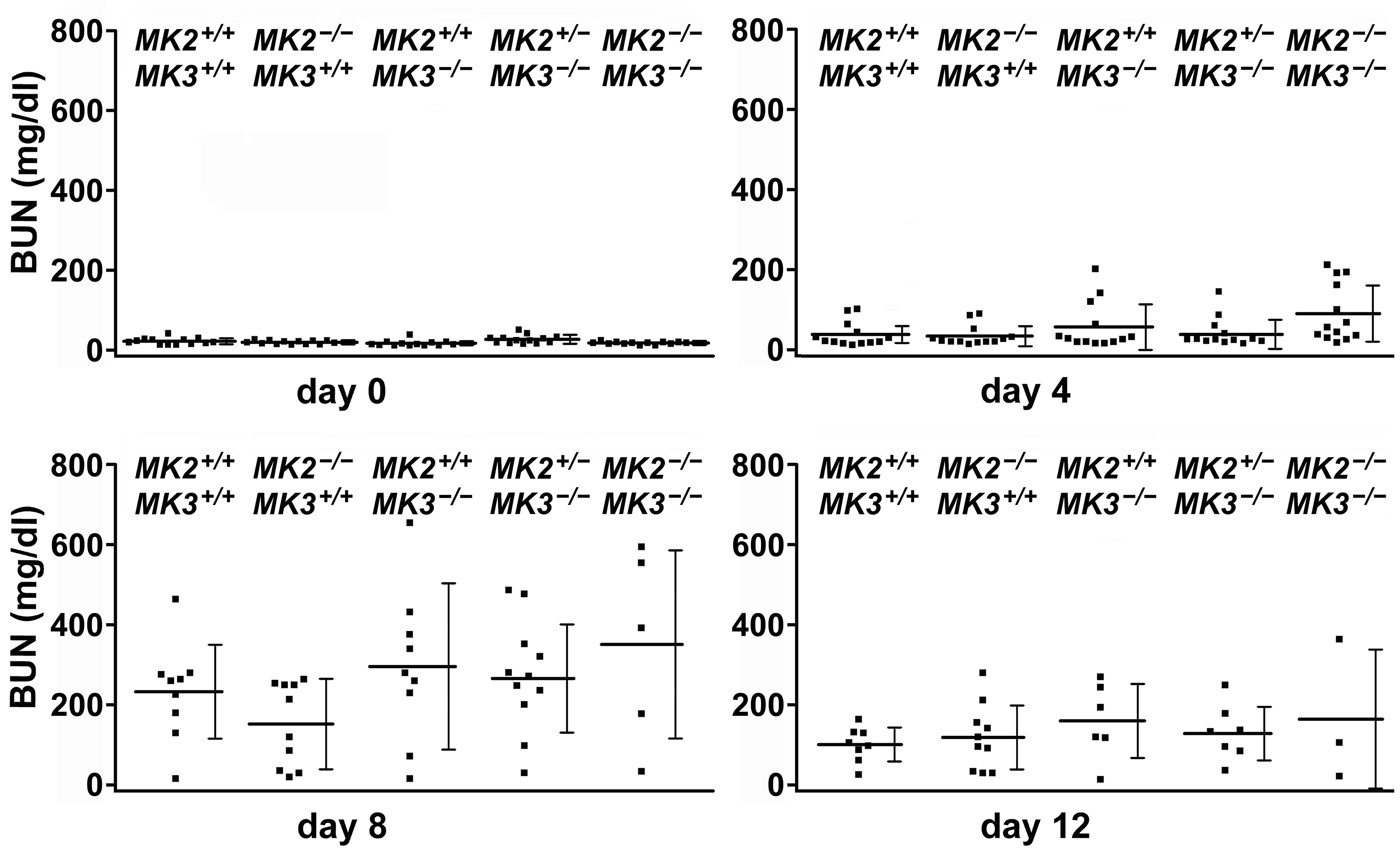

Supplement: Figure S2 — Effect of MK2 and MK3 genotypes on BUN in response to the AMC serum. The BUN values collected at day 0 prior to AMC serum injection and at days 4, 8, and 12 following AMC serum injection were plotted for each surviving mouse. Horizontal bars indicate the means and error bars represent S.D. At days 8 and 12, all means were significantly different from the baseline values at day 0 of the same genotype group. The trend of BUN values was consistent with the proteinuria data, with the MK2/MK3 double knock-out mice being most susceptible to injury. (TIF) [file pone.0054239.s002.tif]
