# Supplementary material for: Crucial Roles of the Protein Kinases MK2 and MK3 in a Mouse Model of Glomerulonephritis
Source: PLoS One. 2013 Jan 23;8(1):e54239. doi: 10.1371/journal.pone.0054239 (PMC3553169; doi:10.1371/journal.pone.0054239)
Supplement: Text S1 — Additional information on mouse genotyping and determinations of blood urea nitrogen, blood creatinine and body weight. (DOC) [file pone.0054239.s003.doc]

**Supporting Information**

**Text S1. Additional information on mouse genotyping and determinations of blood urea nitrogen, blood creatinine and body weight**

**Genotyping of MK2 and MK3 knock-out mice**

Mice were genotyped by PCR analysis of alkaline-lysed toe clips as previously described [1, 2]. Briefly, wild-type alleles were discriminated from knockout alleles using an amplicon that spans the 5’ LoxP insertion site of either gene. MK2 wild-type alleles were detected using a forward (5’-CATGC CATGATGAGGTGCCTCTGC) and a reverse primer (5’-CCCTCTCTACCTCTTTCTGTGAATGCC), while MK2 knock-out alleles were detected using the same forward primer, and a reverse primer (5’-CTGTTGTGCCCAGTCATAGCCG) which is specific for the neomycin resistance cassette. Wild-type and knock-out MK2 alleles produce amplicons of 200 bp and 560 bp, respectively. MK3 wild-type alleles were detected using a forward primer (5’-GCCAATGTCCCGCATTATCTCTGC) and a reverse primer (5’-CAGGGAGCACTCACAGAGCAGTGGGC), while MK3 knock-out alleles were detected using the same forward primer and a reverse primer which is again specific for the neomycin resistance cassette. Wild-type and knock-out MK3 alleles produce amplicons of 879 bp and 1239 bp, respectively. PCR products were resolved on 1.5% agarose gels and visualized by ethidium bromide staining (Figure S1A). In order to verify the various MK2 and MK3 genotypes at the protein level, extracts of renal cortices were analyzed by SDS-PAGE/western blotting for presence of MK2 and MK3 at day 0 (untreated mice) (Figure S1B).

**Blood urea nitrogen**

Blood was collected by puncturing the submandibular vein with a 16-gauge needle and collected in lithium heparin containing tubes with a total volume of 100 µl each.

Blood urea nitrogen (BUN) was measured using the Point of Care I-STAT and 6+ Testing cartridges according to the manufacturer's instruction (Abbott Hematology, Santa Clara, CA).  Blood was first diluted 1:1 with PBS, and 95 µl was applied to the cartridge.  Beginning on day 8, the blood dilutions were 1:4 since the I-STAT cannot measure BUN levels greater than 140 mg/dl.

The effects of the various *MK2* and *MK3* genotypes on BUN values are shown in Figure S2. Baseline BUN values (day 0) in mice with the various genotypes were indistinguishable from those of the wild-type mice, suggesting that deletion of MK2, MK3, or both had no apparent impact on baseline renal function which is consistent with the proteinuria data. In response to AMC serum, the average BUN values increased in mice of all genotypes with a peak at day 8, followed by a decrease at day 12 in the fraction of surviving mice.  This increase was statistically significant across all genotypes, despite the variability among the individual mice within each group.  The AMC serum model of APGN typically manifests this high degree of variability of the BUN values [3].  Given this variability within each group, statistical analyses failed to establish differences between the various MK2 and MK3 knock-out mice and the wild-type mice.  Nevertheless, the trend of BUN values was consistent with the proteinuria data, with the MK2/MK3 double knock-out mice being most susceptible to injury.

**Blood creatinine estimation**

Blood samples were collected as described above and stored at -20^o^C. Changes in whole blood creatinine content over the course of the experiment, relative to day 0, were estimated on a Vitros 5,1 chemistry analyzer (Ortho-Clinical Diagnostics), which measures creatinine enzymatically using creatinine amidinohydrolase. The imprecision was 2.0 and 1.1% at concentrations of 88.4 and 486.2 µmol/l, respectively, thus confirming the accuracy of this method. A standard curve comparing mouse plasma with varying concentrations of creatinine to paired frozen whole blood samples was used to relate whole blood creatinine to plasma creatinine.

We noticed an increase in blood creatinine relative to day 0 in single mice, especially at day 8 following AMC serum injection, however, the average increases in the various experimental groups did not reach statistical significance due to the high degree of variability (not shown).

**Mouse body weight**

In some experimental groups, an increase in mouse body weight at days 8 and 12 was observed which was indicative of renal failure (Table S1). Since the time course of weight gain (maximum at day 12) did not coincide with the peaks of the mortality rate (days 4-6) and renal injury (days 4-12, depending on the genotype), the weight gain was not apparent in all groups. This weight gain was most pronounced (~35%) at day 12 in the MK2 heterozygote MK3 knock-out mice (*MK2^+/–^MK3^–/–^*), which correlated well with the highest proteinuria observed in this group at day 12

**References**

1. Kotlyarov A, Neininger A, Schubert C, Eckert R, Birchmeier C, et al. (1999) MAPKAP kinase 2 is essential for LPS-induced TNF-alpha biosynthesis. Nat Cell Biol 1: 94-97.

2. Ronkina N, Kotlyarov A, Dittrich-Breiholz O, Kracht M, Hitti E et al. (2007) The mitogen-activated protein kinase (MAPK)-activated protein kinases MK2 and MK3 cooperate in stimulation of tumor necrosis factor biosynthesis and stabilization of p38 MAPK. Mol Cell Biol 27: 170-181.

3. Yo Y, Braun MC, Barisoni L, Mobaraki H, Lu H, et al. (2003) Anti-mouse mesangial cell serum induces acute glomerulonephropathy in mice. Nephron Exp Nephrol 93: e92-106.

**Table S1. Body weight of the surviving mice of the various MK2/MK3 knock-out genotypes in the course of the experiment.** The average body weight at day 0 (prior to disease induction) in each experimental group was set at 100%. Alterations in the average body weights over the course of the experiment are expressed as percent of the initial average weight (**±** S.D.). The averages of the absolute weights (in g **±** S.D.) are given in parenthesis. Significant increases from day 0 are indicated by a dagger (**†**).

| **genotype** | **day 0** | **day 4** | **day 8** | **day 12** |
| --- | --- | --- | --- | --- |
| *MK2^+/+^MK3^+/+^* | **100 %** **± 5.3**  (16.0 g ± 0.8) | **100.6 % ± 4.7**  (16.1 g ± 0.8) | **114.6 % ± 5.9** **†**  (18.3 g ± 1.1) | **122.2 % ± 11.8** **†** (19.5 g ± 2.3) |
| *MK2^–/–^MK3^+/+^* | **100 % ± 8.5**  (17.1 g ± 1.4) | **100.0 % ± 7.5**  (17.1 g ± 1.3) | **110.1 % ± 9.2**  (18.8 g ± 1.7) | **108.0 % ± 16.9**  (18.5 g ± 3.1) |
| *MK2^+/+^MK3^–/–^* | **100 % ± 5.6**  (15.7 g ± 0.9) | **102.4 % ± 6.8**  (16.1 g ± 1.1) | **106.1 % ± 8.3**  (16.7 g ± 1.4) | **105.6 % ± 14.6**  (16.6 g ± 2.4) |
| *MK2^+/–^MK3^–/–^* | **100 % ± 5.3**  (15.2 g ± 0.8) | **107.6 % ± 5.4**  (16.4 g ± 0.9) | **120.6 % ± 10.3** **†** (18.4 g ± 1.9) | **134.5 % ± 15.6 †** (20.5 g ± 3.2) |
| *MK2^–/–^MK3^–/–^* | **100 % ± 5.2**  (16.7 g ± 0.9) | **100.1 % ± 5.0**  (16.7 g ± 0.8) | **98.8 % ± 13.6**  (16.5 g ± 2.3) | **102.2 % ± 15.9**  (17.1 g ± 2.7) |
